# Analysis of Patients with Urolithiasis Visiting the Emergency Department between 2014 and 2016 in Korea: Data from the National Emergency Department Information System

**DOI:** 10.1038/s41598-019-52950-8

**Published:** 2019-11-12

**Authors:** Jong Wook Kim, Jung-Youn Kim, Sun Tae Ahn, Mi Mi Oh, Du Geon Moon, Hong Seok Park

**Affiliations:** 1 Department of Urology, College of Medicine, Korea University, Seoul, Korea; 2 Department of Emergency Medicine, College of Medicine, Korea University, Seoul, Korea

**Keywords:** Epidemiology, Urological manifestations

## Abstract

This study investigated the characteristics of patients with urolithiasis visiting an emergency department based on a national database system in Korea. This study spanned a period of three years from January 1, 2014 to December 31, 2016. A retrospective census was conducted using the National Emergency Department Information System for urolithiasis patients. Patient data, including age, sex, insurance type, emergency department visit date and time, discharge date and time, emergency department treatment result, visit flow, and hospitalization route, were extracted and analyzed. Overall, 103,981, 112,083, and 120,647 patients/year during the 2014–2016 study period visited an emergency department with a diagnosis related to urolithiasis. Total monthly emergency department visits ranged from 35,927 in August (highest) to 24,008 in February. Overall, 13.2% of patients were hospitalized and the hospitalization rate was stable (estimated annual percent change) over the study period. Patients aged <9 years or ≥70 years and those with medical aid had higher hospitalization rates. A higher number of visits occurred in the hot season, on weekends, and in the 6 a.m. and 8 p.m. time slots. This nationwide study revealed that the percentage of patients visiting an emergency department with urolithiasis was higher in August, in the early morning, and at weekends.

## Introduction

Patients who visit emergency department (ED) due to acute abdominal pain are often diagnosed with urolithiasis. The chief complaint of patients with urolithiasis is a sudden onset of flank pain, lower back pain to the genitalia, and hematuria^1^. Urolithiasis is a common disease worldwide. The prevalence of urolithiasis in Korea has been reported to be approximately 5.7%^2^. According to literature, the life-time development risk is 12% (in men) and 6% (in women), and the lifetime cumulative incidence ranges from 5–10%^1,3,4^. The 10-year recurrence is high, at approximately 42–50%^5,6^. Most urolithiasis patients receive acute treatment in ED^7,8^.

The causes of urolithiasis are multifactorial. Numerous epidemiological studies indicate sex, race, age, climate, occupation, and obesity influence occurrence^9,10^. Except for urolithiasis patients who visit a hospital for follow-up observation, most patients receive treatment in ED for pain control^7^. Therefore, ED is a very important contact point for treatment of urolithiasis patients. Although the overall prevalence of urolithiasis has been examined previously, few studies have evaluated the characteristics of urolithiasis patients visiting ED. As most urolithiasis patients visit ED, their use of ED should be analyzed to improve treatment and management at limited ED facilities.

In the present study, information was extracted relative to urolithiasis patients from a national database of ED. The data were surveyed and analyzed for epidemiologic characteristics of urolithiasis patients visiting ED over a three-year period. Based on the general characteristics analyzed, the state of use of ED and hospital resources was examined. This study attempted to provide essential data useful for patient treatment and efficient management of limited resources.

## Materials and Methods

### Study design and database

This study utilized National Emergency Department Information System (NEDIS) for secondary data analysis. NEDIS is an emergency information network operated by the government (Ministry of Health & Welfare) since 2003 and is controlled by National Emergency Medical Center. NEDIS includes clinical and administrative data of all patients who have visited ED across the country^11^. In Korea, a national health insurance service is provided that covers approximately 98% of the total population. Thus, national data are considered influential. Emergency centers across the nation undergo approval assessment yearly to be approved as an emergency service institution. Essentially, they are required to digitalize all data items of NEDIS and transmit them for assessment. Thus, it may be assumed that the data used in this study reflect the data of all ED in Korea.

### Data collection

A retrospective census of urolithiasis patients who visited ED over the three-year study period (January 1, 2014 to December 31, 2016) was conducted. Patient data, including age, sex, insurance type, ED visit date and time, discharge date and time, ED treatment outcomes, visit route, and hospitalization path, were extracted from the national database and were analyzed after an official application for access to data. The corresponding ICD codes used for diagnoses are N20, N21, N22 and N23.

### Outcome measures

General characteristics of the patients were compared. Hospitalized patients and discharged patients were examined to analyze their epidemiologic characteristics, such as visit date and time, age, sex, treatment outcome, the number of individual visits by the same patient compared to all ED visits, the length of stay in the ED, hospitalization rate, and main visit duration (hours and months).

### Statistical analyses

For all variables, the hospitalization group was compared to the non-hospitalization group. All statistical analyses were performed using SPSS software (version 20.0; IBM SPSS, Armonk, NY, USA) and Excel (Microsoft Corporation, Redmond, WA, USA). For frequency analysis, a one-way analysis of variance test was conducted. Null hypotheses of no difference were rejected with p-values < 0.05. Data are expressed as n (%), mean ± standard deviation (SD).

### Ethics statement

This research received approval from the institutional review board of Korea University Guro Hospital (No. 2018GR0136). The requirement for informed consent from the participants was waived by the board.

## Results

The number of urolithiasis patients visiting the EDs from 2014–2016 was 336,711. Of these, patients who required hospitalization for treatment accounted for 13.2%, while 86.8% were discharged after treatment. The average age of the patients overall was 47.8 ± 15 years, and hospitalized patients were older on average than those discharged (53.9 ± 17.2 vs. 46.8 ± 14.4 years). Men outnumbered women (222,659 [66.1%] vs. 114,052 [33.9%]) and 11% of men required hospitalization, whereas only 17.3% of women were hospitalized. The hospitalization rate did not differ according to the grade of the emergency center.

Although patients visiting EDs on weekends numbered 110,431 (32.8%), the hospitalization rate was higher on weekdays (13.7% vs. 12.1%). Patients with commercial insurance coverage or a specific insurance type, such as car insurance, had higher hospitalization rates, and patients with Medicaid insurance had a higher hospitalization rate than those who had general Medicare. The number of patients who visited EDs as individual walk-in patients was higher than those transferred from other hospitals or by ambulance. In cases of hospitalization, patients arriving by ambulance outnumbered those arriving by other means. Patients who were hospitalized remained in the ED longer and had a longer time from occurrence to a visit. The number of urolithiasis patients increased steadily per year of the study (103,981 vs. 112,083 vs. 120,647), while the number of patients who visited the nation’s EDs overall also increased constantly per year (8,033,594 vs. 8,512,834 vs. 9,268,112). Urolithiasis patients accounted for 1.3% of those visiting the EDs (Table 1).

**Table 1:** Characteristics of patients diagnosed with urolithiasis in the emergency department according to NEDIS data from 2014 to 2016.

	Total	Admission (%)		Treated and discharged (%)		P value
Patients, n (%)	336,711	44,323	13.2	292,388	86.8	
Age (mean ± SD)	47.8 ± 15.0	53.9 ± 17.2		46.8 ± 14.4		<0.001
Sex n (%):						<0.001
Male	222,659	24,576	11.0	198,083	89.0	
Female	114,052	19,747	17.3	94,305	82.7	
No. ED* type (%):						<0.001
Regional emergency medical center	47,096	5,992	12.7	41,104	87.3	
Sub-specialty emergency medical center	700	114	16.3	586	83.7	
Local emergency medical center	106,756	14,535	13.6	92,221	86.4	
Local emergency medical agency	181,063	23,463	13.0	157,600	87.0	
Unknown	1,096	219	20.0	877	80.0	
Most common diagnosis code:						<0.001
N201 (Calculus of ureter)	262,489	27,104	10.3	235,385	89.7	
N219 (Calculus of lower urinary tract, unspecified)	17,368	1,442	8.3	15,926	91.7	
Patient status:						<0.001
Urgent	257,202	36,586	14.2	220,616	85.8	
Non-urgent	79,480	7,737	9.7	71,743	90.3	
Others	29	0	0.0	29	100.0	
Weekend ED visit, n (%):						<0.001
No	226,280	31,012	13.7	195,268	86.3	
Yes	110,431	13,311	12.1	97,120	87.9	
Type of insurance, n (%)						<0.001
Medicare	320,692	41,226	12.9	279,466	87.1	
Medicaid 1	7,044	2,073	29.4	4,971	70.6	
Medicaid 2	2,437	327	13.4	2,110	86.6	
Other	1,465	170	11.6	1,295	88.4	
Unknown	214	19	8.9	195	91.1	
Private	2	0	0.0	2	100.0	
Commercial	101	54	53.5	47	46.5	
Uninsured	4,456	282	6.3	4,174	93.7	
Car	300	172	57.3	128	42.7	
Route of visit						<0.001
Direct visit	315,792	36,363	11.5	279,429	88.5	
Transferred-in	17,577	6,603	37.6	10,974	62.4	
From outpatient area	2,820	1,319	46.8	1,501	53.2	
Others	522	38	7.3	484	92.7	
Mode of arrival, n (%)						<0.001
Private transportation (car)	260,304	30,419	11.7	229,885	88.3	
Public ambulance service	50,010	7,720	15.4	42,290	84.6	
Walk-in	18,592	2,553	13.7	16,039	86.3	
Private ambulance service	3,277	2,065	63.0	1,212	37	
Other hospital ambulances	1,640	1,103	67.3	537	32.7	
Aeromedical transport	178	23	12.9	155	87.1	
Public transportation (e.g., police car)	167	15	9.0	152	91	
Other	2,543	425	16.7	2,118	83.3	
Length of ED stay	2.6 ± 5.11	5.3 ± 28.1		2.2 ± 5.1		<0.001
Time from onset to ED arrival	18.1 ± 160.4	39.6 ± 160.4		14.9 ± 160.4		<0.001
Year, n (%)						<0.001
2014	103,981 (1.3%)	13,320	12.8	90,661	87.2	
2015	112,083 (1.3%)	14,905	13.3	97,178	86.7	
2016	120,647 (1.3%)	16,098	13.3	104,549	86.7	

*NEDIS*, National Emergency Department Information System.

**Figure 1:**
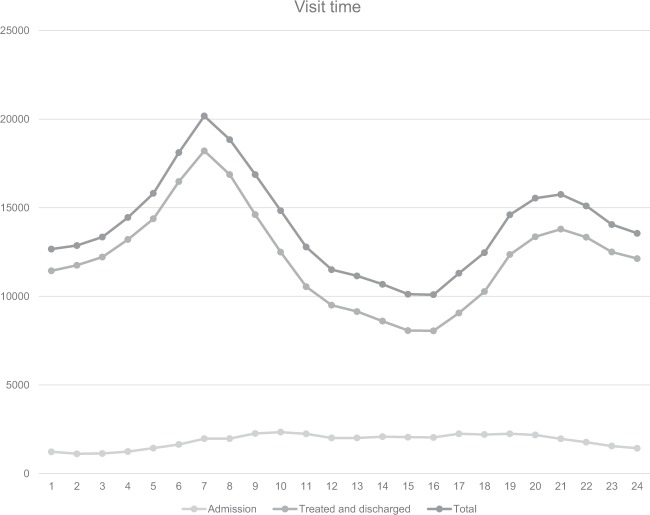
Number of Emergency Department visits by time of day. Total number of ED visits, admissions, treated and discharged patients for urolithiasis in Korea from 2014 to 2016 in the NEDIS stratified by time of day in hours.

A higher number of urolithiasis patients visited the EDs between 6–7 a.m. and 8–10 p.m. Most patients who visited the EDs during daytime were hospitalized (Fig. 1).

**Figure 2:**
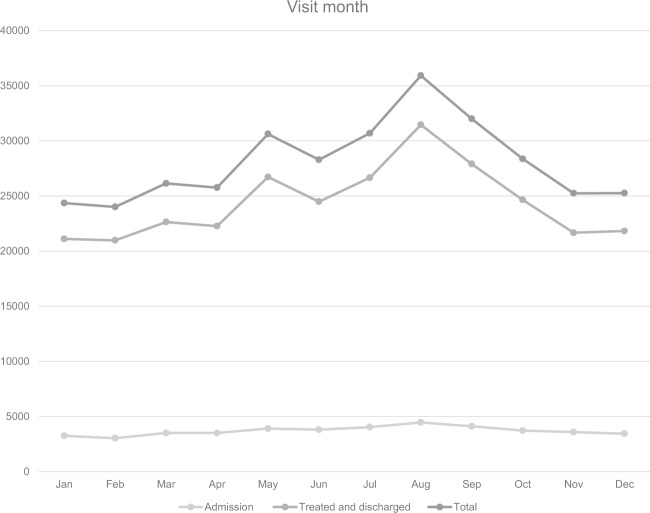
Number of Emergency Department visits by month. Total number of Emergency Department visits, admissions, treated and discharged patients for urolithiasis in Korea from 2014 to 2016 in NEDIS stratified by month.

**Figure 3:**
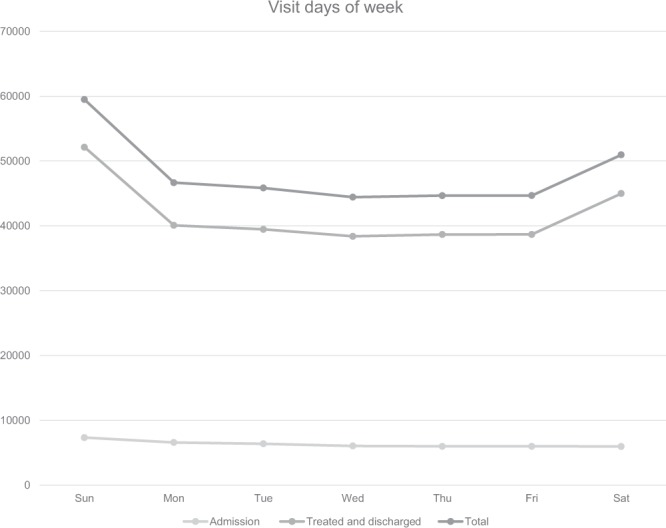
Number of Emergency Department visits subdivided by the day of the week. Total number of Emergency Department visit, admission, treated, and discharged patient for urolithiasis in Korea in 2014 to 2016 in NEDIS stratified by day of the week.

The largest number of patients visited the EDs in August (35,927), followed by September (32,099), July (30,695), and May (30,624). In terms of seasons, the highest visits occurred in summer. February had the fewest visits (24,008) (Fig. 2). The largest number of patient visits occurred on Saturdays and Sundays (Fig. 3).

**Figure 4:**
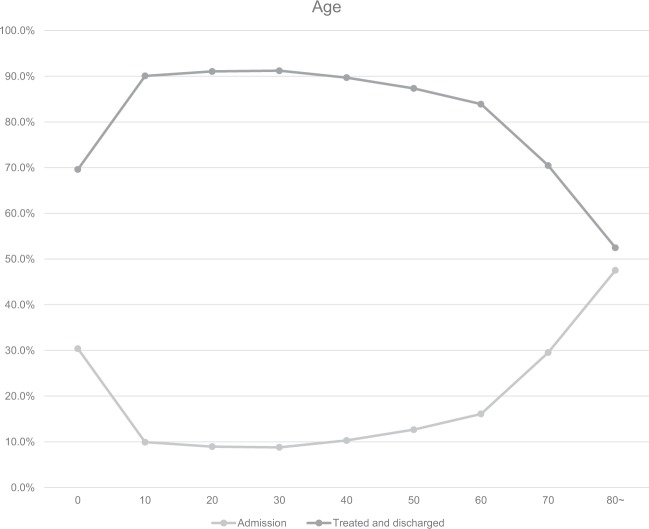
Ratio of Emergency Department visits by age. Ratio of total Emergency Department visits, admission, treated and discharged patients for urolithiasis in Korea from 2014 to 2016 in the NEDIS stratified by age.

Compared to other age groups, patients in their 50s comprised the largest number of patients visiting the EDs, followed by patients in their 40s and 30s (Table 1), while patients <9 years (30.4%) and those in their 70s and 80s and older had a remarkably high hospitalization rate (Fig. 4).

The stay in the ED was 2.6 ± 5.11 hours on average and for hospitalized patients this stay was longer (5.3 ± 28.1 vs. 2.2 ± 5.1).

The annual rate of hospitalized patients was not significantly different (12.8% vs. 13.3% vs. 13.3% from 2014 to 2016, respectively). The number of urolithiasis patients visiting the EDs increased steadily annually from 2014 to 2016 (103,981 vs. 112,083 vs. 120,647); However the proportion of urolithiasis patients among all ED patients remained constant at 1.3% for all three years.

## Discussion

This study is meaningful because it examined the epidemiologic characteristics of urolithiasis patients visiting ED and hospitalized patients in Korea based on a national database. Severe acute flank pain is the most common symptom of urolithiasis. Thus, if urolithiasis occurs, patients are highly likely to visit ED. However, there are few studies about nation-wide information on such visits to ED.

Since NEDIS data includes data from national ED, they are important in terms of analysis of the characteristics of patients visiting Korean ED. The government operated NEDIS includes the clinical and administrative data of all patients visiting ED^11^. The data collected include age, sex, type of insurance, an initial vital signs, the visit flow, date of visit and discharge.

Acute treatment for urolithiasis is mainly aimed at controlling symptoms. Therefore, in cases in which subjective indicators are treated, different factors may influence treatment outcomes and these may subjected to social influences. Thus, medical costs, the type of insurance coverage, and treating hospital, social environment of the hospital visit may influence the patient through different mechanisms and may ultimately lead to a pattern of hospitalization^12^.

This study showed that the number of urolithiasis patients visiting the EDs increased gradually, however the proportion of urolithiasis patients among all ED patients remained constant. Similarly, Roghmann *et al*. reported that the number of ED patients remained stable^13^. Other national studies have reported that the proportion of urolithiasis patients among ED patients was 1.8%, but these were based on a single year; thus, any increase in was impossible to determine^14^. With regard to the prevalence of urinal stones, some studies have reported a gradual increase, with lifetime cumulative incidence reaching up to 37%^4,9,15,16^. Currently, it has been reported that urolithiasis was increasing in Asia due to the influence of westernized culture^4,17^. Taylor *et al*. showed that obesity and metabolic syndrome could influence the occurrence of urolithiasis^10^. Thus, ED visits of urolithiasis patients may differ in terms of country, society, and period; therefore, conducting additional epidemiological research is important.

In this study, the hospitalization rate of urolithiasis patients was approximately 13.2%. Urolithiasis occurred more frequently in men, despite more female patients being hospitalized. In addition, the hospitalization rate of patients aged <9 years was 30.4%, and those aged ≥70 years had a higher hospitalization rate. The annual rate of hospitalized patients was not significantly different. Ghani *et al*.^18^ reported that the hospitalization rate of upper urinary tract stone patients in the United States was 12%, a result that was similar to that of this study. Moreover, the increased trend in hospitalization rate observed in the American study was not significant, which was similar to this study. Although the reasons for which the hospitalization rate remained constant may vary, it may be due to more accurate diagnosis based on non-contrast CT and better pain control of spontaneous stone passage during medical treatment^19–21^. With the rapid universalization of CT images, clinicians are able to receive information on the existence of stones and on their location and size. Such information is considered to be helpful to determine drug treatment and hospitalization.

Korea has four different seasons: spring (March to May), summer (June to August), fall (September to November), and winter (December to February.). Numerous reports have confirmed the seasonal variation of urolithiasis. It is well known that when temperature increases, the discharge of calcium through urine increases, or calcium oxalate or calcium phosphate excessively saturates, and thus the potential for urolithiasis formation increases^22–24^. Furthermore, the frequency of urolithiasis occurrence was higher in the months of August, July, and May in order. Nevertheless, months with higher frequency of occurrence did not correspondingly have higher hospitalization rates. Lin *et al*.^25^, in a study from Taiwan, investigated the relationship between ESWL, the main treatment for urolithiasis, and environmental temperature. The ESWL count showed a strong association with temperature. They also suggested that excessive sweating in hot weather led to a reduction in urinary output and concentration, which resulted in increased stone formation. Sirohi *et al*.^26^ revealed that there was a close relationship with temperature based on an analysis of the changes in monthly temperature in New York.

According to Hong *et al*.^14^, who surveyed patients aged ≥18 years in selected EDs in 2010, a year earlier than the start year of the data used in this study, the average age of the subjects was 45 years, and the male-to-female ratio was 2:1. Most study subjects visited the EDs between 6–10 a.m. and the highest number of visits occurred in August. The main symptoms reported by the study subjects were flank pain, abdominal pain, and hematuria in order. The average stay in the ED was 171 hours and women remained longer than men. Park *et al*.^27^ also revealed that women visited EDs twice as often than men, just as in this study. Most studies have shown that the prevalence of urolithiasis was higher in men and had a hospitalization rate of approximately 6%–8%, which differed from the results of this study (13.2%), a difference likely attributable to the lack of young children <18 years included in the study. Indeed, the hospitalization rate of children aged <9 years among ED patients was high (30.4%). In this study, the stay in the ED was 2.6 ± 11.3 hours on average and for hospitalized patients this stay was longer, which was likely influenced by patients whose main indication for hospitalization of urolithiasis was uncontrolled pain. The indication for hospitalization occurs when a patient’s pain worsens in the ED despite different attempts at its alleviation. Such patients will remain in the ED longer than patients whose pain improves and return home after treatment. Bae *et al*.^28^ reported that upper urinary stones were found most often among patients in their 40s. In our study, the number of ED patients in their 40s was the highest. In addition, according to this study, most visits occurred at 7 a.m., and a further peak was observed at 9 p.m. (Fig. 1). However, the hospitalization rate of ED patients following daytime visits was higher. A larger number of ED visits occurred in the hotter season, at 7 a.m., in men, on weekends, and by those in their 50s; however, the hospitalization rate of such groups was not higher than that of other groups. Instead, a higher hospitalization rate occurred in November, and comprised women, on weekday visits, and for patients <9 years old and for those aged ≥70 years.

This study has some limitations. First, patients with a diagnosis defined as simple abdomen were not subjected to additional examination and were likely to be excluded. Patient data were extracted basis on the diagnostic definition given. For this reason, patients whose symptoms only were descriptive may not have received a definitive diagnosis. Nevertheless, patients who were definitely diagnosed with urolithiasis through additional examinations, rather than clinical diagnosis alone, were more likely to have received a specific diagnosis definition in the database. Therefore, few urolithiasis patients appeared to have been omitted. Secondly, despite the database-based extraction, there were few clinical data registered regarding diagnostic modalities, individual treatments or prognosis. Therefore, further clinical research will be necessary. Finally, the authors did not look into factors that lead to hospitalization, the results should be interpreted with caution.

## Conclusion

This is the first study to analyze the characteristics of urolithiasis patients visiting ED based on a national database comprising patients of all age groups. Urolithiasis patients accounted for 1.3% of all patients who visited ED and of these, those requiring hospitalization for treatment accounted for 13.2%. Female patients had a higher hospitalization rate than males. Of patients aged <9 years and those aged ≥70 years older, those with medical insurance had a higher hospitalization rate. More visits occurred in hot seasons, on weekends, and in the 6 a.m. and 8 p.m. slots. The characteristics of urolithiasis patients who visited an ED described by this study will help shape the treatment approach for these patients in an ED and will help guide the management of ED resources. It is necessary to continue collecting basic patient data from those who visit domestic medical centers.
